# Imbalance of type I and II interferon pathways is associated with pain perception in Sjogren’s disease: a real-life study

**DOI:** 10.3389/fimmu.2026.1778090

**Published:** 2026-04-02

**Authors:** Krzysztof Proc, Paweł Gajdanowicz, Lucyna Korman, Maciej Wuczyński, Marek Jutel, Piotr Wiland, Maciej Sebastian, Marta Madej, Magdalena Zemelka-Wiacek, Agata Sebastian

**Affiliations:** 1 Department of Rheumatology and Internal Medicine, Institute of Internal Medicine, Wroclaw Medical University, Wroclaw, Poland; 2 Department of Clinical Immunology, Faculty of Medicine, Wroclaw Medical University, Wroclaw, Poland; 3 Statistical Analysis Centre, Wroclaw Medical University, Wroclaw, Poland; 4 University Centre of General and Oncological Surgery, Wroclaw Medical University, Wroclaw, Poland

**Keywords:** interferon balance, interferons, pain, Sjögren’s disease, systemic lupus erythematosus, type I interferon, type III interferon

## Abstract

**Introduction:**

Activation of interferon (IFN) pathways is a central mechanism in the pathogenesis of Sjögren’s disease (SjD) and systemic lupus erythematosus (SLE). While type I IFN signatures have been extensively characterized, the systemic relevance of circulating type III IFNs and the clinical implications of the relative balance between IFN types I, II, and III remain insufficiently explored.

**Methods:**

In this real-life, exploratory study, plasma concentrations of IFN-α2, IFN-β, IFN-γ, IFN-λ1, and IFN-λ2 were measured in patients with SjD (n = 59), SLE (n = 35), and healthy controls (n = 30). Disease activity and patient-reported outcomes were assessed using ESSDAI, ESSPRI, and SLEDAI-2K. In addition to absolute IFN concentrations, ratios between IFN subtypes were analyzed to reflect interferon pathway balance. Non-parametric statistical analyses with correction for multiple testing were applied.

**Results:**

Absolute plasma concentrations of individual IFN subtypes did not differ significantly between the study groups. In SjD, exploratory correlation analyses revealed a significant association between the IFN-α2/IFN-γ ratio and pain severity assessed by ESSPRI (Spearman’s ρ = 0.48, adjusted *p* < 0.05). This association was independent of systemic inflammatory activity measured by ESSDAI. Furthermore, selected IFN ratios involving IFN-λ differed between SLE and SjD, suggesting disease-specific patterns of interferon balance. No significant associations were observed between IFN parameters and SLEDAI-2K scores in SLE.

**Conclusions:**

These findings indicate that the relative balance between interferon pathways, rather than absolute circulating IFN levels, may be clinically relevant in SjD, particularly with respect to patient-reported pain. Interferon subtype ratios may represent exploratory biomarkers of immune dysregulation. Further studies integrating systemic, tissue-level, and transcriptomic approaches are warranted to validate these observations.

## 1 Introduction

Sjögren’s disease (SjD) and systemic lupus erythematosus (SLE) are systemic autoimmune diseases characterized by chronic immune activation and heterogeneous clinical manifestations (1–3). A central element of their pathogenesis is the activation of interferon (IFN) signaling pathways, particularly type I interferons, which promote dendritic cell maturation, B-cell activation and autoantibody production. Persistent activation of IFN-induced genes, referred to as the “interferon signature”, has been consistently demonstrated in both SjD and SLE (1, 4–7).

While the role of type I IFNs has been extensively studied, less is known about the contribution of type II (IFN-γ) and type III interferons (IFN-λ). IFN-γ is a key mediator of Th1-driven immune responses, whereas IFN-λ plays an important role in epithelial and mucosal immunity. Emerging evidence suggests that IFN-λ expression is increased locally in minor salivary glands of SjD patients; however, its systemic relevance and relationship with clinical manifestations remain unclear (7, 8).

Importantly, growing data indicate that immune-mediated symptoms such as pain, fatigue and dryness are not always directly linked to objective inflammatory activity (2, 3, 7). This raises the possibility that qualitative dysregulation of immune signaling—such as imbalance between IFN pathways—may contribute to patient-reported disease burden. We therefore hypothesized that the relative balance between IFN types I, II and III, rather than their absolute circulating levels, may be associated with clinical activity and subjective symptoms in SjD and SLE.

The aim of this exploratory, real-life study was to assess circulating IFN profiles and IFN subtype ratios in SjD and SLE patients and to evaluate their associations with disease activity, particular emphasis on pain perception.

## 2 Materials and methods

### 2.1 Study population

The study included 59 patients with SjD, 35 patients with SLE and 30 age- and sex-matched healthy controls. Healthy controls were recruited among healthcare workers (physicians and nurses) from the Department of Rheumatology and Internal Medicine All patients fulfilled the 2016 ACR/EULAR classification criteria for primary SjD and the 2019 EULAR/ACR criteria for SLE (3, 9). Exclusion criteria comprised age <18 years, active or recent infection, malignancy, immunodeficiency, diabetes, recent vaccination, or treatment with interferon-targeted or JAK–STAT pathway inhibitors.

### 2.2 Clinical assessment

In all patients, after obtaining written informed consent, demographic and clinical data were collected for all patients. Disease activity was evaluated using validated scales and questionnaires:

- For patients with SjD, the ESSDAI (10) (assessing organ involvement) and ESSPRI (11) (assessing severity of dryness, pain, and fatigue over the preceding 2 weeks; 0 mm = minimum, 100 mm = maximum symptom severity) were used;
- For patients with SLE, disease activity was assessed using the SLEDAI-2K questionnaire (12);
- In both SjD and SLE patients, the following laboratory parameters were measured: antinuclear antibodies (ANA), complete blood count, complement components C3 and C4, rheumatoid factor (RF), C-reactive protein (CRP), erythrocyte sedimentation rate (ESR), serum immunoglobulin levels (IgG, IgM, IgA), gammaglobulins, and cryoglobulins.

Plasma samples were collected and stored at −80 °C for subsequent determination of the interferon panel, including IFN-α2, IFN-λ1, IFN-λ2, IFN-β and IFN-γ. Serum interferon concentrations were evaluated using the LEGENDplex™ assay (BioLegend, LEGENDplex™ Human Type 1/2/3 Interferon Panel, 5-plex). The analysis was performed using a BD CANTO II flow cytometer and the LEGENDplex™ data analysis software suite, according to the manufacturer’s manual.

Detection levels for plasma concentration of interferons were:

- IFN-λ1 - 1525.36,
- IFN-α2 - 154.95,
- IFN-λ2 - 2120.1,
- IFN-β - 519.6,
- IFN-γ - 292.87.

The study was conducted in accordance with the principles of the Helsinki Declaration and was approved by the local Bioethics Committee (Decision No. KB- 57/2024). Written informed consent was obtained from all participants. The study was carried out with funds allocated as part of a subsidy for the maintenance and development of the research potential of the Medical University of Wroclaw, Poland (SUBZ.A270.24.083).

### 2.3 Statistical analysis

Data distribution was presented as medians and quartiles. To compare values of parameters in SjD, SLE and control groups, Kruskal-Wallis test was performed. To assess relationships between target variables and IFNs, Spearman correlation test was used. As target variables were non-continuous, it was necessary to apply non-parametric tests. In order to address multiple testing problem, Bonferroni correction was applied. In each set of correlation tests, all p-values were multiplied by 15. All values below detection levels were removed from the data. In order to verify robustness of the results, statistical tests were repeated twice using two modified datasets. The first modified dataset included only pharmacologically treated patients from experimental groups. The second modified dataset did not exclude values below detection levels. Instead, single value imputation was performed using detection thresholds divided by square root of two (13). All calculations were performed using Python 3.15.5 programming language and SciPy 1.15.3 package (14). Statistical significance was set at α = 0.05 for all tests.

## 3 Results

### 3.1 Basic characteristics of the study groups (SjD and SLE)

SjD group comprised 55 women and 4 men, with a mean age of 57 years (range 22–78). The mean disease duration was 7 years (range 0.5–30). The mean ESSDAI score was 3.5 points (range 0–36mm), and the mean ESSPRI scores were 62 mm for dryness (range 8–100 mm), 53 mm for fatigue (range 0–87 mm), and 47 mm for pain (range 0–89 mm). All patients were positive for ANA, including anti-SSA in 50 patients, anti-Ro52 in 48, anti-SSB in 28, AMA-M2 in 8, anti-centromere in 3, anti-Jo1 in 2, and anti-Sm/RNP in 2 patients. Regarding therapy, 42 patients received hydroxychloroquine (100–400 mg/day), 16 received prednisone at a daily dose of 2.5–10 mg, 10 received methotrexate (10–25 mg/week), 2 received azathioprine (50–100 mg/day), and 5 received mycophenolate mofetil (1–2 g/day). Compared with SLE patients, SjD patients more frequently exhibited elevated RF levels, whereas hypocomplementemia (C3 and/or C4) was significantly less common. No other differences were observed between the two groups regarding complete blood count, CRP, or immunoglobulin levels (**Tables 1**, **2**).

**Table 1:** Baseline characteristics of patients with Sjögren’s disease (SjD), systemic lupus erythematosus (SLE), and healthy controls.

Parameter	SjD (n = 59)	SLE (n = 35)	Healthy controls (n = 30)	*p*-value
Female/Male, n	55/4	31/4	27/3	>0.05
Mean age, years	57	47	44	>0.05
Leukocytes (nv: 4–10 ×10³/µl)				
– mean value	5.5	6.5	N/A	>0.05
– leukopenia, n	9	6	N/A	>0.05
Lymphocytes (nv: 1.5–4.5 ×10³/µl)				
– mean value	1.8	1.4	N/A	>0.05
– lymphopenia, n	38	23	N/A	>0.05
Platelets (nv: 150–400 ×10³/µl)				
– mean value	244	266	N/A	>0.05
– thrombocytopenia, n	4	3	N/A	>0.05
Hemoglobin (nv: 12–16 g/dl)				
– mean value	12.9	12.6	N/A	>0.05
– anemia, n	11	11	N/A	>0.05
Hypocomplementemia C3 (nv:0.82–1.85 g/l) or C4 (nv0.15–0.53 g/l), n	1	33	N/A	<0.05
CRP (nv: 0–5 mg/l)				
– mean value	3.3	3.4	N/A	>0.05
– increased, n	10	6	N/A	>0.05
ESR (nv: 1–10 mm/h)				
– mean value	23	21	N/A	>0.05
– increased, n	42	27	N/A	>0.05
Serum IgG (nv: 5.4–18.22 g/l)				
– mean value	15	14	N/A	>0.05
– increased, n	11	4	N/A	>0.05
Serum IgM (nv: 0.22–2.4 g/l)				
– mean value	1.3	1.2	N/A	>0.05
– increased, n	4	2	N/A	>0.05
Serum IgA (nv: 0.63–4.84 g/l)				
– mean value	2.8	2.6	N/A	>0.05
– increased, n	4	3	N/A	>0.05
Gammaglobulins (electrophoresis; nv: 7–14 g/l)				
– mean value	13.2	12.8	N/A	>0.05
– hypergammaglobulinemia, n	14	6	N/A	>0.05
Rheumatoid factor (RF) (nv: 0–30 IU/ml)				
– mean value	110.2	32.5	N/A	<0.05
–increased, n	33	3	N/A	<0.05
Cryoglobulins (nv:present), n	2	0	N/A	N/A

n-number of patients; N/A- not applicable; RF- rheumatoid factor; nv- normal range of value.

**Table 2:** Medians and quartiles of target and predictor variables for three study groups.

Characteristic / study group	SjD	SLE	Control
ESSDAI	2.0 (0.0, 3.0)	N/A	
ESSPRI dryness	62.0 (45.0, 80.5)	N/A	
ESSPRI fatigue	59.0 (37.5, 68.0)	N/A	
ESSPRI pain	50.0 (25.0, 73.0)	N/A	
SLEDAI2K	N/A	4.0 (2.0, 7.5)	
IFN- λ1	2301.26 (2048.32, 2533.62)	1999.04 (1702.48, 2327.72)	2351.36 (2042.79, 2501.89)
IFN-α2	504.09 (416.37, 647.38)	510.84 (367.54, 616.36)	609.27 (456.3, 655.43)
IFN- λ2	3317.4 (2641.3, 4091.3)	2969.9 (0.0, 3547.45)	3410.8 (3105.98, 3637.08)
IFN- β	813.92 (634.42, 1055.78)	885.66 (653.96, 1385.78)	861.16 (717.91, 1022.44)
IFN- γ	487.25 (354.0, 621.04)	464.66 (0.0, 597.04)	479.53 (395.87, 623.57)
IFN- λ1/IFN-α2	4.49 (3.65, 5.59)	4.3 (3.38, 4.87)	3.89 (3.51, 4.23)
IFN- λ1/IFN- λ2	0.64 (0.58, 0.75)	0.7 (0.59, 0.75)	0.69 (0.63, 0.71)
IFN- λ1/IFN- β	2.54 (2.31, 3.11)	2.3 (1.47, 2.77)	2.45 (2.07, 3.09)
IFN- λ1/IFN- γ	4.12 (3.64, 5.1)	3.89 (3.16, 4.33)	4.28 (3.79, 4.9)
IFN-α2/IFN- λ2	0.16 (0.13, 0.19)	0.17 (0.13, 0.22)	0.17 (0.16, 0.19)
IFN-α2/IFN- β	0.64 (0.5, 0.79)	0.52 (0.4, 0.69)	0.66 (0.52, 0.77)
IFN-α2/IFN- γ	1.01 (0.85, 1.23)	1.05 (0.87, 1.34)	1.13 (0.98, 1.32)
IFN- λ2/IFN- β	3.97 (3.33, 4.77)	3.04 (1.86, 3.89)	3.84 (3.12, 4.6)
IFN- λ2/IFN- γ	6.46 (5.46, 7.37)	5.65 (5.19, 6.92)	6.51 (5.74, 7.67)
IFN- β/IFN- γ	1.65 (1.22, 1.97)	1.63 (1.45, 3.41)	1.71 (1.48, 1.99)

N/A – not applicable, SjD – Sjögren’s disease, SLE – systemic lupus erythematosus.

The SLE group included 31 women and 4 men, with a mean age of 47 years (range 19–72). The mean disease duration was 10 years (range 0.5–32). The mean SLEDAI-2K score was 5 points (range 0–19). All patients were ANA-positive, including anti-dsDNA in 17 patients, anti-Ro52 in 14, and anti-Sm/RNP in 12 patients. Regarding therapy, 30 patients received hydroxychloroquine (200–400 mg/day), 28 received prednisone at a mean daily dose of 2.5–40 mg, 7 received methotrexate (15–25 mg/week), 3 received azathioprine (100–150 mg/day), and 9 received mycophenolate mofetil (500 mg–2 g/day).

### 3.2 Interferon profiling

The highest concentrations of interferons α, γ, and λ1–2 were detected in the healthy control group, whereas IFN-β levels were highest in patients with systemic lupus erythematosus (SLE). In patients with SjD, interferon concentrations consistently exhibited intermediate values between those observed in SLE patients and healthy controls. Importantly, none of the observed differences between groups reached statistical significance (**Tables 2**, **3**).

**Table 3:** Mean concentrations of individual interferon subtypes in patients with Sjögren’s disease (SjD), systemic lupus erythematosus (SLE), and healthy controls.

Type of interferon	SjD	SLE	Healthy controls	p-value
IFN-α2	540	530	618	>0.05
IFN-β	831	1082	607	>0.05
IFN- γ	539	409	562	>0.05
IFN- λ1	2263	1909	2296	>0.05
IFN- λ2	3220	2563	3315	>0.05

SjD, Sjögren disease; SLE, systemic lupus erythematosus; IFN, interferon; p-value statistically significant <0.005.

**Figure 1:**
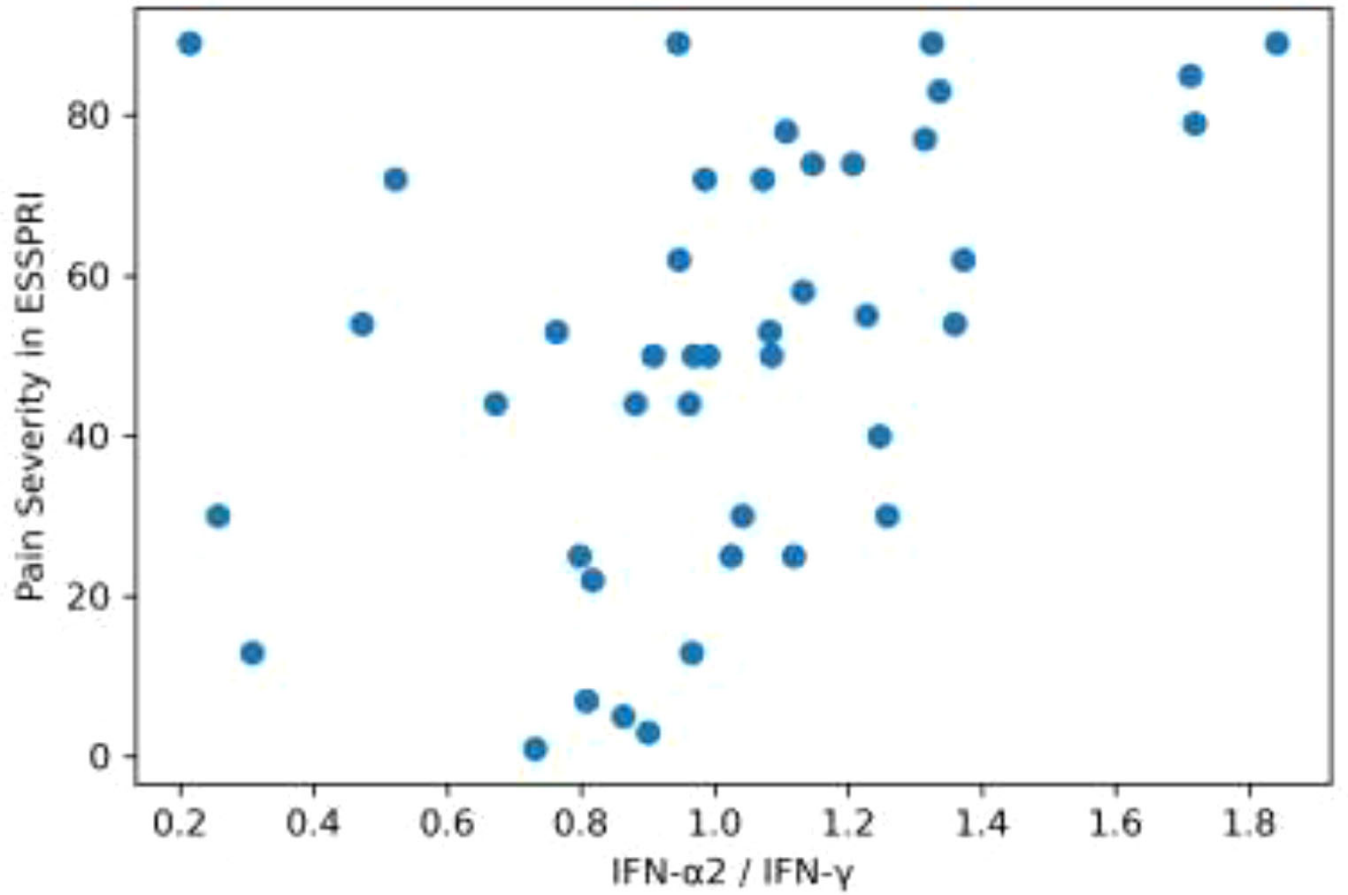
Scatterplot depicting a correlation between IFN-α2/IFN-γ ratio and pain severity in ESSPRI (Spearman’s ρ = 0.48).

A weak association (Spearman’s ρ between 0.33 and 0.36) was identified between IFN-λ2 and ESSDAI, IFN-λ1/IFN-λ2 and ESSDAI, as well as between IFN-λ2/IFN-γ and IFN-β/IFN-γ ratios and pain severity assessed by ESSPRI. In contrast, a strong association (Spearman’s ρ = 0.48) was observed between the IFN-α2/IFN-γ ratio and pain severity in ESSPRI (**Figure 1**). No associations were detected between interferon levels and SLEDAI-2K scores in patients with SLE.

The IFN-λ1/IFN-β ratio was significantly higher in the SLE group, whereas the IFN-λ2/IFN-β ratio was significantly lower in SLE patients. No additional significant associations were identified in the SjD, SLE, or healthy control groups. Interferon concentrations and correlation analyses across all three groups are summarized in **Tables 2**, **3** and **Supplementary Tables S1-S6** (**Supplementary Table S1**-**S6** are provided in the **Supplementary Material**).

Additional analysis was performed after excluding all patients that did not receive any pharmacological treatment. This excluded 9 patients from SjD group, 1 patient from SLE group and all patients from control group. Results from this analysis confirmed previous findings (**Supplementary Tables S7**-**S12**). Another analysis was done after imputing interferon concentrations below limit of detection (LOD) with LOD/√2. **Table 4** presents the number of samples that have been imputed. The results confirmed all findings of the core analysis except different levels of IFN- λ1/IFN-β ratio between study groups (**Supplementary Tables S13**-**S18**).

**Table 4:** Counts and percentages of samples in the study groups that fallen below the detection limit and were imputed in the additional analysis.

Type of interferon	SjD	SLE	Healthy controls	All patients
IFN-α2	1 (1.69%)	3 (8.57%)	1 (3.33%)	5 (4.03%)
IFN-β	8 (13.56%)	7 (20.00%)	1 (3.33%)	16 (12.90%)
IFN- γ	11 (18.64%)	12 (34.29%)	4 (13.33%)	27 (21.77%)
IFN- λ1	2 (3.39%)	4 (11.43%)	1 (3.33%)	7 (5.65%)
IFN- λ2	8 (13.56%)	10 (28.57%)	4 (13.33%)	22 (17.74%)

## 4 Discussion

The mean values of the analyzed interferon subtypes did not differ between patients with Sjögren’s disease (SjD), systemic lupus erythematosus (SLE), and the healthy control group. However, novel observations emerged from the analysis of interferon associations and ratios. A weak relationship was identified between IFN-α2 and ESSDAI, IFN-λ1/IFN-λ2 and ESSDAI, as well as between IFN-λ2/IFN-γ and IFN-β/IFN-γ ratios and pain severity assessed by ESSPRI. A strong relationship was observed between the IFN-α2/IFN-γ ratio and pain severity in ESSPRI. No associations were observed between interferon levels and SLEDAI-2K in SLE.

Higher IFN-λ1/IFN-β ratios were observed in the SLE group, whereas IFN-λ2/IFN-β ratios were lower in this group. Relationships between different types of interferons (e.g., IFN-α2/IFN-γ, IFN-β/IFN-γ) suggest a newly identified disproportion between type I interferons (α, β) and type II interferon (γ) in SjD. The observed association between the IFN-α2/IFN-γ ratio and perceived pain in SjD may suggest that the balance between type I and type II interferon axes could be relevant forpain perception and subjective symptoms, independently of inflammatory activity measured by ESSDAI.

Given the number of interferon subtype ratios analyzed and the relatively limited sample size, the present study should be considered exploratory. Although Bonferroni correction was applied to reduce the risk of false-positive findings, its conservative nature may have increased the risk of false-negative results. The observed association between the IFN-α2/IFN-γ ratio and pain severity may represent a potential link between interferon pathway balance and pain perception in SjD; however, this observation should be regarded as exploratory and hypothesis-generating. The proposed hypothesis linking pain severity to the IFN-α2/IFN-γ ratio requires confirmation in larger independent cohorts.

IFN-λ represents a relatively poorly explored area in SjD. Previous studies have shown that the expression of IFN-λ and its receptor (IL28RA/IFN-λR1) in minor salivary glands of patients with SjD was significantly higher than in healthy individuals with sicca symptoms, suggesting local involvement of IFN-λ in exocrine glands (15). Although IFN-λ expression is increased locally, it remains unclear whether modulation of this pathway (e.g., administration or blockade of IFN-λ) provides clinical benefit.

Sjögren’s disease is primarily a tissue-driven disorder involving salivary glands and peripheral nerves, where local interferon activity may be more directly relevant to disease mechanisms than circulating cytokine levels. Circulating interferon concentrations and interferon subtype ratios should therefore be interpreted as surrogate biomarkers of interferon pathway activation and indirect indicators of local immune activity rather than direct measures of interferon activity within affected tissues. Further studies integrating systemic and tissue-level analyses are needed to clarify these relationships.

Based on the present findings, it may be cautiously inferred that IFN-λ2 could be involved at the systemic level in SjD. However, this observation should be regarded as a preliminary hypothesis requiring confirmation in further well-designed studies.

Furthermore, the disproportion between type I/III and type II interferons (IFN-λ2/IFN-γ and IFN-β/IFN-γ) may increase pain sensitivity.

A weak association between IFN-α2 concentration and the disease activity index ESSDAI suggests that activation of the type I interferon (IFN-I) axis may contribute to the severity of systemic manifestations of the disease. IFN-α2, as a representative of IFN-I, plays a key role in the activation of both innate and adaptive immune responses, including stimulation of B lymphocytes and autoantibody production. Therefore, increased IFN-α2 levels may reflect a higher degree of immune activation and inflammation, translating into increased clinical activity as assessed by ESSDAI. The higher the IFN-α2 level, the more active the disease course—although this association is moderate, which may indicate that type I interferon is one of, but not the sole, factors influencing disease activity.

These findings are partly inconsistent with results published by Bodewes et al. (4). The authors demonstrated that patients with IFN-I or IFN-I+II activity exhibited higher IgG levels and more frequent anti-SSA/SSB antibodies; however, no differences in the total ESSDAI score were observed between groups. Only the biological domain of ESSDAI was higher in patients with an active interferon pathway. In certain patient subgroups, a low IFN-I signature (i.e., reduced expression of interferon-stimulated genes) may occur, for example in individuals with disease limited to the salivary and lacrimal glands, without systemic manifestations (16).

In contrast, our results are consistent with two other studies (6, 7). In the first, an IFN-I signature in monocytes was identified in approximately 55% of patients with SjD. Its presence was associated with disease activity (including the cutaneous domain of ESSDAI) and higher BAFF expression (6). In the second study, IFN-α levels were shown to be associated with clinical and immunological features of SjD, as well as with more frequent systemic complications during a 5-year follow-up (7).

In our analysis, we did not observe dominance of individual interferon subtypes in SjD or SLE. However, we identified a statistically significantly higher IFN-λ1/IFN-β ratio in the SLE group and a lower IFN-λ2/IFN-β ratio. This indicates a relative predominance of IFN-λ1 compared with IFN-β in patients with SLE. IFN-λ1 may therefore be excessively active in SLE and may contribute to chronic immune system activation, whereas IFN-λ2 may be insufficiently active or its production may be impaired during the disease course. To date, elevated IFN-λ1 levels have been reported in SLE and shown to correlate with disease activity (SLEDAI) and manifestations such as nephropathy and arthritis (17, 18). However, IFN-λ1 and IFN-λ2 subtypes and their relationship to IFN-β have not previously been analyzed simultaneously, as performed in the present study. The lack of additional interferon-related associations, including in SjD, points to the complex role of these pathways and the involvement of other mechanisms driving disease activity.

One unexpected finding of our study was the pronounced interferon activation observed in the healthy control group. The interpretation of these findings requires particular caution. The control group consisted of healthy healthcare workers (physicians and nurses) from the Department of Rheumatology and Internal Medicine who were regularly exposed to patients with chronic inflammatory and infectious diseases, including pneumonia, COVID-19, influenza and other respiratory viral infections. Such occupational exposure may have contributed to increased baseline interferon activity.

Detailed information on recent infections in the control group was not available. During the study period approximately 20% of controls received seasonal influenza vaccination and fewer than 5% received a COVID-19 booster dose.

Interpretation of circulating interferon concentrations is further complicated by the substantial biological variability of cytokine levels. Baseline cytokine and interferon concentrations may vary widely between healthy individuals and over time; therefore, single time-point measurements should be interpreted with caution (18).

In addition, lower interferon levels observed in patients with SjD and SLE may have been influenced by ongoing immunosuppressive therapy, including hydroxychloroquine and other immunosuppressive treatments known to inhibit interferon production and signaling (7, 19). Furthermore, interferon activation in Sjögren’s disease is heterogeneous, and circulating interferon levels may vary substantially between patients (1).

Another possible explanation is immune exhaustion associated with chronic autoimmune activation. Persistent immune stimulation in long-standing inflammatory diseases may lead to altered interferon responses and reduced cytokine production capacity (20).

Therefore, this observation represents an important limitation affecting the interpretation of absolute interferon concentrations.

The limitations of this study include the lack of measurements of individual IFN-α subtypes, where potentially relevant differences may be masked, as individual isoforms may differ in immunomodulatory profiles, and selective activity across different cell types. In addition, as in many real-life studies, it was not possible to fully eliminate the potential influence of ongoing therapies on the obtained results.

Another limitation concern differences between treatments which could bias IFN levels. Individual patients were treated using different types of pharmaceutics and very small group of patients in experimental groups (9 in SjD group and 1 in SLE group) were not pharmacologically treated. This fragmentation of data hindered our ability to perform a stratified analysis, allowing only to compare a subset of patients that received any kind of pharmacological treatment.

## 5 Summary

Our results confirm that activation of the interferon axis, particularly type I and type III interferons, plays an important role in the pathogenesis of SjD. Increased expression of IFN-λ and its receptor in salivary glands, together with the association between IFN-λ2 and the severity of subjective symptoms (ESSPRI), points to local involvement of this pathway in the inflammatory process. The correlation between IFN-α2 and disease activity (ESSDAI) further supports the role of type I interferons in amplifying immune responses, in line with previous observations. Differences in IFN-λ1/IFN-β and IFN-λ2/IFN-β ratios between SjD and SLE suggest distinct regulation of these pathways in the two diseases. These findings underscore the complexity of interferon-mediated mechanisms and highlight the need for further studies addressing their clinical relevance and therapeutic potential in SjD. Additionally, the observed differences between IFN-λ1 and IFN-λ2 suggest that individual type III interferon subtypes may exert distinct roles in the regulation of autoimmunity in SLE.

Taken together, our findings suggest that interferon subtype ratios may better reflect clinically relevant immune dysregulation than absolute interferon concentrations in SjD.

## Data Availability

The raw data supporting the conclusions of this article will be made available by the authors, without undue reservation.
