# Supplementary material for: Imbalance of type I and II interferon pathways is associated with pain perception in Sjogren’s disease: a real-life study
Source: Front Immunol. 2026 Apr 2;17:1778090. doi: 10.3389/fimmu.2026.1778090 (PMC13107005; doi:10.3389/fimmu.2026.1778090)
Supplement: Supplementary file 1 [file Table1.docx]

Supplementary Material

# Supplementary Tables

Supplementary Table 1. Results of Kruskal-Wallis test for three study groups. First column present number of valid observations in SjD, SLE and Control groups. Second column show values of H statistic resulting from Kruskal-Wallis test. The last column show p-value of the result. Statistically significant results were marked with an asterisk.

|  | n | H | p |
| --- | --- | --- | --- |
| IFN- λ1 | [57, 31, 29] | 4.93 | 0.085 |
| IFN-α2 | [58, 32, 29] | 3.03 | 0.219 |
| IFN- λ2 | [51, 25, 26] | 1.02 | 0.602 |
| IFN-β | [51, 28, 29] | 2.88 | 0.237 |
| IFN- γ | [48, 23, 26] | 0.53 | 0.766 |
| IFN- λ1 / IFN-α2 | [56, 30, 29] | 4.03 | 0.133 |
| IFN- λ1 / IFN- λ2 | [49, 23, 26] | 0.40 | 0.820 |
| IFN- λ1 / IFN-β | [50, 26, 28] | 6.17 | 0.046* |
| IFN- λ1 / IFN- γ | [46, 22, 26] | 1.95 | 0.378 |
| IFN-α2 / IFN- λ2 | [50, 25, 26] | 2.86 | 0.239 |
| IFN-α2 / IFN-α2 | [50, 27, 28] | 3.96 | 0.138 |
| IFN-α2 / IFN- γ | [48, 23, 26] | 2.92 | 0.232 |
| IFN- λ2 / IFN-β | [46, 23, 25] | 6.82 | 0.033* |
| IFN- λ2 / IFN- γ | [45, 22, 24] | 2.09 | 0.352 |
| IFN-β / IFN- γ | [45, 22, 25] | 1.62 | 0.444 |

Supplementary Table 2. Results of Spearman correlation test for ESSDAI target variable for SjD study group. Values on the diagonal present test results for the five IFNs. Values outside the diagonal show correlations between IFN proportions and ESSDAI variable. P-values were multiplied 15 times in order to remove the effect of multiple testing.

|  | IFN- λ1 | IFN-α2 | IFN- λ2 | IFN-β | IFN- γ |
| --- | --- | --- | --- | --- | --- |
| IFN- λ1 | n = 41 rho = -0.12 p = 1.000 | n = 41 rho = 0.08 p = 1.000 | n = 33 rho = 0.36 p = 0.597 | n = 35 rho = 0.24 p = 1.000 | n = 33 rho = 0.0 p = 1.000 |
| IFN-α2 |  | n = 43 rho = -0.13 p = 1.000 | n = 35 rho = 0.13 p = 1.000 | n = 36 rho = 0.09 p = 1.000 | n = 35 rho = -0.12 p = 1.000 |
| IFN- λ2 |  |  | n = 35 rho = -0.35 p = 0.559 | n = 31 rho = 0.1 p = 1.000 | n = 32 rho = -0.19 p = 1.000 |
| IFN-β |  |  |  | n = 36 rho = -0.27 p = 1.000 | n = 32 rho = -0.11 p = 1.000 |
| IFN- γ |  |  |  |  | n = 35 rho = -0.12 p = 1.000 |

Supplementary Table 3. Results of Spearman correlation test for ESSPRI-dryness target variable for SjD study group. Values on the diagonal present test results for the five IFNs. Values outside the diagonal show correlations between IFN proportions and ESSPRI-dryness variable. P-values were multiplied 15 times in order to remove the effect of multiple testing.

|  | IFN- λ1 | IFN-α2 | IFN- λ2 | IFN-β | IFN- γ |
| --- | --- | --- | --- | --- | --- |
| IFN- λ1 | n = 57 rho = -0.01 p = 1.000 | n = 56 rho = -0.12 p = 1.000 | n = 49 rho = -0.19 p = 1.000 | n = 50 rho = -0.05 p = 1.000 | n = 46 rho = -0.13 p = 1.000 |
| IFN-α2 |  | n = 58 rho = 0.09 p = 1.000 | n = 50 rho = 0.03 p = 1.000 | n = 50 rho = 0.17 p = 1.000 | n = 48 rho = 0.07 p = 1.000 |
| IFN- λ2 |  |  | n = 51 rho = 0.23 p = 1.000 | n = 46 rho = 0.09 p = 1.000 | n = 45 rho = 0.05 p = 1.000 |
| IFN-β |  |  |  | n = 51 rho = 0.04 p = 1.000 | n = 45 rho = -0.11 p = 1.000 |
| IFN- γ |  |  |  |  | n = 48 rho = 0.13 p = 1.000 |

Supplementary Table 4. Results of Spearman correlation test for ESSPRI-fatigue target variable for SjD study group. Values on the diagonal present test results for the five IFNs. Values outside the diagonal show correlations between IFN proportions and ESSPRI-fatigue variable. P-values were multiplied 15 times in order to remove the effect of multiple testing.

|  | IFN- λ1 | IFN-α2 | IFN- λ2 | IFN-β | IFN- γ |
| --- | --- | --- | --- | --- | --- |
| IFN- λ1 | n = 55 rho = -0.02 p = 1.000 | n = 54 rho = -0.08 p = 1.000 | n = 48 rho = 0.02 p = 1.000 | n = 48 rho = -0.01 p = 1.000 | n = 44 rho = 0.0 p = 1.000 |
| IFN-α2 |  | n = 56 rho = 0.04 p = 1.000 | n = 49 rho = 0.07 p = 1.000 | n = 48 rho = 0.1 p = 1.000 | n = 46 rho = 0.24 p = 1.000 |
| IFN- λ2 |  |  | n = 50 rho = -0.03 p = 1.000 | n = 45 rho = 0.06 p = 1.000 | n = 44 rho = 0.12 p = 1.000 |
| IFN-β |  |  |  | n = 49 rho = -0.04 p = 1.000 | n = 43 rho = 0.09 p = 1.000 |
| IFN- γ |  |  |  |  | n = 46 rho = -0.08 p = 1.000 |

Supplementary Table 5. Results of Spearman correlation test for ESSPRI-pain target variable for SjD study group. Values on the diagonal present test results for the five IFNs. Values outside the diagonal show correlations between IFN proportions and ESSPRI-pain variable. P-values were multiplied 15 times in order to remove the effect of multiple testing. Significant result was marked with an asterisk.

|  | IFN- λ1 | IFN-α2 | IFN- λ2 | IFN-β | IFN- γ |
| --- | --- | --- | --- | --- | --- |
| IFN- λ1 | n = 51 rho = -0.05 p = 1.000 | n = 50 rho = -0.24 p = 1.000 | n = 44 rho = -0.1 p = 1.000 | n = 45 rho = -0.19 p = 1.000 | n = 41 rho = 0.23 p = 1.000 |
| IFN-α2 |  | n = 52 rho = 0.18 p = 1.000 | n = 45 rho = 0.04 p = 1.000 | n = 45 rho = 0.1 p = 1.000 | n = 43 rho = 0.48 p = 0.017* |
| IFN- λ2 |  |  | n = 46 rho = 0.01 p = 1.000 | n = 42 rho = -0.08 p = 1.000 | n = 41 rho = 0.34 p = 0.443 |
| IFN-β |  |  |  | n = 46 rho = 0.04 p = 1.000 | n = 40 rho = 0.33 p = 0.586 |
| IFN- γ |  |  |  |  | n = 43 rho = -0.2 p = 1.000 |

Supplementary Table 6. Results of Spearman correlation test for SLEDAI2K target variable for SLE study group. Values on the diagonal present test results for the five IFNs. Values outside the diagonal show correlations between IFN proportions and SLEDAI2K variable. P-values were multiplied 15 times in order to remove the effect of multiple testing. Significant result was marked with an asterisk.

|  | IFN- λ1 | IFN-α2 | IFN- λ2 | IFN-β | IFN- γ |
| --- | --- | --- | --- | --- | --- |
| IFN- λ1 | n = 26 rho = -0.16 p = 1.000 | n = 25 rho = -0.27 p = 1.000 | n = 20 rho = 0.24 p = 1.000 | n = 22 rho = -0.31 p = 1.000 | n = 18 rho = -0.03 p = 1.000 |
| IFN-α2 |  | n = 26 rho = 0.05 p = 1.000 | n = 21 rho = 0.11 p = 1.000 | n = 23 rho = -0.15 p = 1.000 | n = 19 rho = -0.04 p = 1.000 |
| IFN- λ2 |  |  | n = 21 rho = -0.13 p = 1.000 | n = 20 rho = -0.2 p = 1.000 | n = 19 rho = -0.26 p = 1.000 |
| IFN-β |  |  |  | n = 24 rho = 0.17 p = 1.000 | n = 18 rho = 0.16 p = 1.000 |
| IFN- γ |  |  |  |  | n = 19 rho = -0.02 p = 1.000 |

Supplementary Table 7. Results of Kruskal-Wallis test for two experimental groups, comparing only pharmacologically treated patients. First column present number of valid observations in SjD and SLE groups. Second column show values of H statistic resulting from Kruskal-Wallis test. The last column show p-value of the result. Statistically significant results were marked with an asterisk.

|  | n | H | p |
| --- | --- | --- | --- |
| IFN- λ1 | [48, 30] | 3.61 | 0.057 |
| IFN-α2 | [49, 31] | 0.05 | 0.828 |
| IFN- λ2 | [42, 24] | 0.24 | 0.622 |
| IFN-β | [43, 27] | 3.00 | 0.083 |
| IFN- γ | [40, 22] | 0.03 | 0.871 |
| IFN- λ1 / IFN-α2 | [47, 29] | 0.69 | 0.407 |
| IFN- λ1 / IFN- λ2 | [40, 22] | 0.00 | 0.988 |
| IFN- λ1 / IFN-β | [42, 25] | 6.00 | 0.014* |
| IFN- λ1 / IFN- γ | [38, 21] | 0.70 | 0.401 |
| IFN-α2 / IFN- λ2 | [41, 24] | 0.91 | 0.341 |
| IFN-α2 / IFN-α2 | [42, 26] | 2.18 | 0.140 |
| IFN-α2 / IFN- γ | [40, 22] | 0.78 | 0.377 |
| IFN- λ2 / IFN-β | [38, 22] | 6.02 | 0.014* |
| IFN- λ2 / IFN- γ | [37, 21] | 0.37 | 0.544 |
| IFN-β / IFN- γ | [37, 21] | 2.49 | 0.115 |

Supplementary Table 8. Results of Spearman correlation test for ESSDAI target variable for SjD study group, including only pharmacologically treated patients. Values on the diagonal present test results for the five IFNs. Values outside the diagonal show correlations between IFN proportions and ESSDAI variable. P-values were multiplied 15 times in order to remove the effect of multiple testing.

|  | IFN- λ1 | IFN-α2 | IFN- λ2 | IFN-β | IFN- γ |
| --- | --- | --- | --- | --- | --- |
| IFN- λ1 | n = 35 rho = -0.11 p = 1.000 | n = 35 rho = 0.12 p = 1.000 | n = 27 rho = 0.49 p = 0.148 | n = 29 rho = 0.17 p = 1.000 | n = 27 rho = -0.03 p = 1.000 |
| IFN-α2 |  | n = 37 rho = -0.15 p = 1.000 | n = 29 rho = 0.16 p = 1.000 | n = 30 rho = -0.02 p = 1.000 | n = 29 rho = -0.18 p = 1.000 |
| IFN- λ2 |  |  | n = 29 rho = -0.46 p = 0.190 | n = 25 rho = -0.02 p = 1.000 | n = 26 rho = -0.28 p = 1.000 |
| IFN-β |  |  |  | n = 30 rho = -0.16 p = 1.000 | n = 26 rho = -0.01 p = 1.000 |
| IFN- γ |  |  |  |  | n = 29 rho = -0.08 p = 1.000 |

Supplementary Table 9. Results of Spearman correlation test for ESSPRI-dryness target variable for SjD study group, including only pharmacologically treated patients. Values on the diagonal present test results for the five IFNs. Values outside the diagonal show correlations between IFN proportions and ESSPRI-dryness variable. P-values were multiplied 15 times in order to remove the effect of multiple testing.

|  | IFN- λ1 | IFN-α2 | IFN- λ2 | IFN-β | IFN- γ |
| --- | --- | --- | --- | --- | --- |
| IFN- λ1 | n = 48 rho = -0.03 p = 1.000 | n = 47 rho = -0.09 p = 1.000 | n = 40 rho = -0.12 p = 1.000 | n = 42 rho = -0.23 p = 1.000 | n = 38 rho = -0.12 p = 1.000 |
| IFN-α2 |  | n = 49 rho = 0.07 p = 1.000 | n = 41 rho = 0.09 p = 1.000 | n = 42 rho = 0.03 p = 1.000 | n = 40 rho = 0.11 p = 1.000 |
| IFN- λ2 |  |  | n = 42 rho = 0.13 p = 1.000 | n = 38 rho = -0.09 p = 1.000 | n = 37 rho = 0.05 p = 1.000 |
| IFN-β |  |  |  | n = 43 rho = 0.18 p = 1.000 | n = 37 rho = 0.03 p = 1.000 |
| IFN- γ |  |  |  |  | n = 40 rho = 0.09 p = 1.000 |

Supplementary Table 10. Results of Spearman correlation test for ESSPRI-fatigue target variable for SjD study group, including only pharmacologically treated patients. Values on the diagonal present test results for the five IFNs. Values outside the diagonal show correlations between IFN proportions and ESSPRI-fatigue variable. P-values were multiplied 15 times in order to remove the effect of multiple testing.

|  | IFN- λ1 | IFN-α2 | IFN- λ2 | IFN-β | IFN- γ |
| --- | --- | --- | --- | --- | --- |
| IFN- λ1 | n = 47 rho = 0.03 p = 1.000 | n = 46 rho = -0.06 p = 1.000 | n = 40 rho = -0.06 p = 1.000 | n = 41 rho = 0.07 p = 1.000 | n = 37 rho = 0.05 p = 1.000 |
| IFN-α2 |  | n = 48 rho = 0.05 p = 1.000 | n = 41 rho = -0.02 p = 1.000 | n = 41 rho = 0.18 p = 1.000 | n = 39 rho = 0.27 p = 1.000 |
| IFN- λ2 |  |  | n = 42 rho = 0.05 p = 1.000 | n = 38 rho = 0.24 p = 1.000 | n = 37 rho = 0.2 p = 1.000 |
| IFN-β |  |  |  | n = 42 rho = -0.07 p = 1.000 | n = 36 rho = 0.06 p = 1.000 |
| IFN- γ |  |  |  |  | n = 39 rho = -0.11 p = 1.000 |

Supplementary Table 11. Results of Spearman correlation test for ESSPRI-pain target variable for SjD study group, including only pharmacologically treated patients. Values on the diagonal present test results for the five IFNs. Values outside the diagonal show correlations between IFN proportions and ESSPRI-pain variable. P-values were multiplied 15 times in order to remove the effect of multiple testing. Significant result was marked with an asterisk.

|  | IFN- λ1 | IFN-α2 | IFN- λ2 | IFN-β | IFN- γ |
| --- | --- | --- | --- | --- | --- |
| IFN- λ1 | n = 47 rho = -0.02 p = 1.000 | n = 46 rho = -0.26 p = 1.000 | n = 40 rho = -0.12 p = 1.000 | n = 41 rho = -0.28 p = 1.000 | n = 37 rho = 0.22 p = 1.000 |
| IFN-α2 |  | n = 48 rho = 0.23 p = 1.000 | n = 41 rho = 0.03 p = 1.000 | n = 41 rho = 0.05 p = 1.000 | n = 39 rho = 0.5 p = 0.020* |
| IFN- λ2 |  |  | n = 42 rho = 0.05 p = 1.000 | n = 38 rho = -0.12 p = 1.000 | n = 37 rho = 0.36 p = 0.465 |
| IFN-β |  |  |  | n = 42 rho = 0.14 p = 1.000 | n = 36 rho = 0.4 p = 0.249 |
| IFN- γ |  |  |  |  | n = 39 rho = -0.19 p = 1.000 |

Supplementary Table 12. Results of Spearman correlation test for SLEDAI2K target variable for SLE study group, including only pharmacologically treated patients. Values on the diagonal present test results for the five IFNs. Values outside the diagonal show correlations between IFN proportions and SLEDAI2K variable. P-values were multiplied 15 times in order to remove the effect of multiple testing. Significant result was marked with an asterisk.

|  | IFN- λ1 | IFN-α2 | IFN- λ2 | IFN-β | IFN- γ |
| --- | --- | --- | --- | --- | --- |
| IFN- λ1 | n = 25 rho = -0.21 p = 1.000 | n = 24 rho = -0.26 p = 1.000 | n = 19 rho = 0.22 p = 1.000 | n = 21 rho = -0.3 p = 1.000 | n = 17 rho = 0.04 p = 1.000 |
| IFN-α2 |  | n = 25 rho = 0.03 p = 1.000 | n = 20 rho = 0.13 p = 1.000 | n = 22 rho = -0.11 p = 1.000 | n = 18 rho = 0.09 p = 1.000 |
| IFN- λ2 |  |  | n = 20 rho = -0.13 p = 1.000 | n = 19 rho = -0.21 p = 1.000 | n = 18 rho = -0.16 p = 1.000 |
| IFN-β |  |  |  | n = 23 rho = 0.17 p = 1.000 | n = 17 rho = 0.24 p = 1.000 |
| IFN- γ |  |  |  |  | n = 18 rho = -0.1 p = 1.000 |

Supplementary Table 13. Results of Kruskal-Wallis test for three study groups, with values below Limit of Detection (LOD) imputed with LOD/√2. First column present number of valid observations in SjD, SLE and Control groups. Second column show values of H statistic resulting from Kruskal-Wallis test. The last column show p-value of the result. Statistically significant results were marked with an asterisk.

|  | n | H | p |
| --- | --- | --- | --- |
| IFN- λ1 | [59, 35, 30] | 7.25 | 0.027 |
| IFN-α2 | [59, 35, 30] | 3.14 | 0.208 |
| IFN- λ2 | [59, 35, 30] | 4.12 | 0.128 |
| IFN-β | [59, 35, 30] | 0.57 | 0.752 |
| IFN- γ | [59, 35, 30] | 1.22 | 0.544 |
| IFN- λ1 / IFN-α2 | [59, 35, 30] | 2.70 | 0.259 |
| IFN- λ1 / IFN- λ2 | [59, 35, 30] | 0.81 | 0.669 |
| IFN- λ1 / IFN-β | [59, 35, 30] | 4.08 | 0.130 |
| IFN- λ1 / IFN- γ | [59, 35, 30] | 0.07 | 0.967 |
| IFN-α2 / IFN- λ2 | [59, 35, 30] | 2.00 | 0.367 |
| IFN-α2 / IFN-α2 | [59, 35, 30] | 2.79 | 0.247 |
| IFN-α2 / IFN- γ | [59, 35, 30] | 1.09 | 0.579 |
| IFN- λ2 / IFN-β | [59, 35, 30] | 6.37 | 0.041* |
| IFN- λ2 / IFN- γ | [59, 35, 30] | 0.07 | 0.968 |
| IFN-β / IFN- γ | [59, 35, 30] | 3.95 | 0.139 |

Supplementary Table 14. Results of Spearman correlation test for ESSDAI target variable for SjD study group, with values below Limit of Detection (LOD) imputed with LOD/√2. Values on the diagonal present test results for the five IFNs. Values outside the diagonal show correlations between IFN proportions and ESSDAI variable. P-values were multiplied 15 times in order to remove the effect of multiple testing.

|  | IFN- λ1 | IFN-α2 | IFN- λ2 | IFN-β | IFN- γ |
| --- | --- | --- | --- | --- | --- |
| IFN- λ1 | n = 43 rho = -0.14 p = 1.000 | n = 43 rho = 0.07 p = 1.000 | n = 43 rho = 0.2 p = 1.000 | n = 43 rho = 0.06 p = 1.000 | n = 43 rho = -0.06 p = 1.000 |
| IFN-α2 |  | n = 43 rho = -0.13 p = 1.000 | n = 43 rho = 0.01 p = 1.000 | n = 43 rho = -0.02 p = 1.000 | n = 43 rho = -0.16 p = 1.000 |
| IFN- λ2 |  |  | n = 43 rho = -0.24 p = 1.000 | n = 43 rho = -0.05 p = 1.000 | n = 43 rho = -0.19 p = 1.000 |
| IFN-β |  |  |  | n = 43 rho = -0.11 p = 1.000 | n = 43 rho = -0.05 p = 1.000 |
| IFN- γ |  |  |  |  | n = 43 rho = -0.04 p = 1.000 |

Supplementary Table 15. Results of Spearman correlation test for ESSPRI-dryness target variable for SjD study group, with values below Limit of Detection (LOD) imputed with LOD/√2. Values on the diagonal present test results for the five IFNs. Values outside the diagonal show correlations between IFN proportions and ESSPRI-dryness variable. P-values were multiplied 15 times in order to remove the effect of multiple testing.

|  | IFN- λ1 | IFN-α2 | IFN- λ2 | IFN-β | IFN- γ |
| --- | --- | --- | --- | --- | --- |
| IFN- λ1 | n = 59 rho = -0.01 p = 1.000 | n = 59 rho = -0.15 p = 1.000 | n = 59 rho = -0.14 p = 1.000 | n = 59 rho = 0.09 p = 1.000 | n = 59 rho = -0.01 p = 1.000 |
| IFN-α2 |  | n = 59 rho = 0.11 p = 1.000 | n = 59 rho = 0.01 p = 1.000 | n = 59 rho = 0.26 p = 0.652 | n = 59 rho = 0.16 p = 1.000 |
| IFN- λ2 |  |  | n = 59 rho = 0.16 p = 1.000 | n = 59 rho = 0.18 p = 1.000 | n = 59 rho = 0.1 p = 1.000 |
| IFN-β |  |  |  | n = 59 rho = -0.1 p = 1.000 | n = 59 rho = -0.1 p = 1.000 |
| IFN- γ |  |  |  |  | n = 59 rho = 0.01 p = 1.000 |

Supplementary Table 16. Results of Spearman correlation test for ESSPRI-fatigue target variable for SjD study group, with values below Limit of Detection (LOD) imputed with LOD/√2. Values on the diagonal present test results for the five IFNs. Values outside the diagonal show correlations between IFN proportions and ESSPRI-fatigue variable. P-values were multiplied 15 times in order to remove the effect of multiple testing.

|  | IFN- λ1 | IFN-α2 | IFN- λ2 | IFN-β | IFN- γ |
| --- | --- | --- | --- | --- | --- |
| IFN- λ1 | n = 57 rho = -0.06 p = 1.000 | n = 57 rho = -0.11 p = 1.000 | n = 57 rho = 0.06 p = 1.000 | n = 57 rho = -0.03 p = 1.000 | n = 57 rho = 0.03 p = 1.000 |
| IFN-α2 |  | n = 57 rho = 0.04 p = 1.000 | n = 57 rho = 0.15 p = 1.000 | n = 57 rho = 0.12 p = 1.000 | n = 57 rho = 0.27 p = 0.641 |
| IFN- λ2 |  |  | n = 57 rho = -0.1 p = 1.000 | n = 57 rho = 0.0 p = 1.000 | n = 57 rho = 0.05 p = 1.000 |
| IFN-β |  |  |  | n = 57 rho = -0.04 p = 1.000 | n = 57 rho = 0.08 p = 1.000 |
| IFN- γ |  |  |  |  | n = 57 rho = -0.09 p = 1.000 |

Supplementary Table 17. Results of Spearman correlation test for ESSPRI-pain target variable for SjD study group, with values below Limit of Detection (LOD) imputed with LOD/√2. Values on the diagonal present test results for the five IFNs. Values outside the diagonal show correlations between IFN proportions and ESSPRI-pain variable. P-values were multiplied 15 times in order to remove the effect of multiple testing. Significant result was marked with an asterisk.

|  | IFN- λ1 | IFN-α2 | IFN- λ2 | IFN-β | IFN- γ |
| --- | --- | --- | --- | --- | --- |
| IFN- λ1 | n = 53 rho = -0.06 p = 1.000 | n = 53 rho = -0.18 p = 1.000 | n = 53 rho = -0.13 p = 1.000 | n = 53 rho = -0.16 p = 1.000 | n = 53 rho = 0.2 p = 1.000 |
| IFN-α2 |  | n = 53 rho = 0.12 p = 1.000 | n = 53 rho = 0.01 p = 1.000 | n = 53 rho = 0.04 p = 1.000 | n = 53 rho = 0.39 p = 0.054 |
| IFN- λ2 |  |  | n = 53 rho = 0.05 p = 1.000 | n = 53 rho = -0.01 p = 1.000 | n = 53 rho = 0.34 p = 0.201 |
| IFN-β |  |  |  | n = 53 rho = 0.04 p = 1.000 | n = 53 rho = 0.31 p = 0.352 |
| IFN- γ |  |  |  |  | n = 53 rho = -0.21 p = 1.000 |

Supplementary Table 18. Results of Spearman correlation test for SLEDAI2K target variable for SLE study group, with values below Limit of Detection (LOD) imputed with LOD/√2. Values on the diagonal present test results for the five IFNs. Values outside the diagonal show correlations between IFN proportions and SLEDAI2K variable. P-values were multiplied 15 times in order to remove the effect of multiple testing. Significant result was marked with an asterisk.

|  | IFN- λ1 | IFN-α2 | IFN- λ2 | IFN-β | IFN- γ |
| --- | --- | --- | --- | --- | --- |
| IFN- λ1 | n = 28 rho = -0.22 p = 1.000 | n = 28 rho = -0.24 p = 1.000 | n = 28 rho = 0.03 p = 1.000 | n = 28 rho = -0.44 p = 0.305 | n = 28 rho = -0.16 p = 1.000 |
| IFN-α2 |  | n = 28 rho = 0.03 p = 1.000 | n = 28 rho = 0.02 p = 1.000 | n = 28 rho = -0.23 p = 1.000 | n = 28 rho = -0.09 p = 1.000 |
| IFN- λ2 |  |  | n = 28 rho = -0.07 p = 1.000 | n = 28 rho = -0.37 p = 0.796 | n = 28 rho = -0.21 p = 1.000 |
| IFN-β |  |  |  | n = 28 rho = 0.28 p = 1.000 | n = 28 rho = 0.27 p = 1.000 |
| IFN- γ |  |  |  |  | n = 28 rho = 0.03 p = 1.000 |
